# Application of Supercritical Solvent Impregnation for Production of Zeolite Modified Starch-Chitosan Polymers with Antibacterial Properties

**DOI:** 10.3390/molecules25204717

**Published:** 2020-10-15

**Authors:** Jelena Pajnik, Ivana Lukić, Jelena Dikić, Jelena Asanin, Milan Gordic, Dusan Misic, Irena Zizović, Malgorzata Korzeniowska

**Affiliations:** 1 Innovation Center of the Faculty of Technology and Metallurgy, University of Belgrade, 11000 Belgrade, Serbia; jpajnik@tmf.bg.ac.rs (J.P.); jdikic@tmf.bg.ac.rs (J.D.); jelenaasanin@gmail.com (J.A.); 2 Faculty of Technology and Metallurgy, University of Belgrade, 11000 Belgrade, Serbia; ilukic@tmf.bg.ac.rs; 3 Vinča Institute of Nuclear Sciences, University of Belgrade, 11351 Vinča, Serbia; milangordic@yahoo.com; 4 Faculty of Veterinary Medicine, University of Belgrade, 11000 Belgrade, Serbia; 5 Faculty of Biotechnology and Food Science, Wroclaw University of Environmental and Life Sciences, 51-651 Wroclaw, Poland; malgorzata.korzeniowska@upwr.edu.pl; 6 Faculty of Chemistry, Wroclaw University of Science and Technology, 50-373 Wroclaw, Poland; irena.zizovic@pwr.edu.pl

**Keywords:** supercritical impregnation, starch-chitosan-zeolite, antibacterial activity

## Abstract

In the present study, supercritical solvent impregnation (SSI) has been applied to incorporate thymol into bio-composite polymers as a potential active packaging material. Thymol, a natural component with a proven antimicrobial activity, was successfully impregnated into starch-chitosan (SC) and starch-chitosan-zeolite (SCZ) films using supercritical carbon dioxide (scCO_2_) as a solvent. Experiments were performed at 35 °C, pressures of 15.5 and 30 MPa, and an impregnation time in the range of 4–24 h. The highest impregnation yields of SC films with starch to chitosan mass ratios of 1:1 and 1:2 were 10.80% and 6.48%, respectively. The addition of natural zeolite (15–60%) significantly increased the loading capacity of films enabling thymol incorporation in a quantity of 16.7–27.3%. FTIR and SEM analyses were applied for the characterization of the films. Mechanical properties and water vapor permeability of films before and after the impregnation were tested as well. Thymol release kinetics in deionized water was followed and modeled by the Korsmeyer-Peppas and Weibull model. SCZ films with thymol loading of approximately 24% exhibited strong antibacterial activity against *E. coli* and methicillin-resistant *Staphylococcus* (S.) *aureus* (MRSA).

## 1. Introduction

Active packaging is an emerging food technology developed to meet constant worldwide market expansion as well as continuous changes in consumers’ demands [1]. Active packaging refers to the improved properties of a packing system designed to provide prolonged shelf life, enhanced quality, and safety of food products [2]. This can be accomplished by the incorporation of active compounds, such as moisture absorbent substances or substances with antimicrobial and antioxidant activity into the food packaging material [2]. One of the most common approaches for packaging films with improved antimicrobial properties is based on the release of active compounds from the packaging material into the close surroundings [3]. Due to stricter standards nowadays in the food industry and the ban on the usage of many substances that were previously allowed in food production [4], natural biodegradable polymers have the advantage of being used for active packaging in comparison to synthetic materials. For the same reasons, instead of synthetic chemicals, mostly molecules of natural origin are preferred to deliver the activity to the food packaging material. Thymol is a colorless, crystalline substance, found in essential oils of thyme, oregano and winter savory with proven antimicrobial effects against Gram-positive and Gram-negative bacteria, and also against some parasites [5,6]. Since it has been “generally recognized as safe” (GRAS) by the Food and Drug Administration (FDA), thymol was found to be a promising bioactive agent for active packaging purposes [2]. Polypropylene [1,3,7] and low-density polyethylene films [8] were impregnated with thymol by the melt blending technique, whereas antibacterial, antifungal and antioxidant effects of the obtained materials were proven. Moreover, following the trends of sustainable development, eco-friendly films and coatings prepared from polysaccharides, proteins and lipids were found to possess superior properties in terms of biodegradability, edibility and biocompatibility compared to conventional synthetic packaging [9]. Starch and chitosan as natural polymers have shown great potential as packaging materials [9,10,11]. The published data show that the presence of thyme essential oil (0.5%, 1% and 2%) on the inner surface of chitosan films significantly reduces the total number of yeast populations [10]. Besides, thyme extract incorporated into the starch-chitosan composite film by the solvent casting method improved its antibacterial and antioxidant properties [9,11].

Zeolites are crystalline, chemically inert, hydrated aluminosilicate porous materials with a large surface and their application has many biological, economic and environmental benefits. Aluminium (Al^3+^) and silicon (Si^4+^) cations connected via oxygen atoms make the zeolite lattice negatively charged. Thus, the other cations such as sodium (Na^+^), potassium (K^+^), calcium (Ca^2+^) or magnesium (Mg^2+^) are also present in the channels and cavities to enable electro neutrality. These cations are mobile and easily exchangeable with other ions [12]. The clinoptilolite, natural type of zeolite, has been proven safe for oral consumption in humans [13,14]. It has been successfully applied in pharmacy due to its pronounced adsorption affinity for different particles and chemicals, including toxins, drugs and microorganisms, as well as the capability for their controlled release [15,16,17]. Besides, modified forms of the natural zeolite exhibited an antacid [18], antidiarrheic [14] antibacterial and anti-inflammatory effect [16]. The clinoptilolite types of zeolite were also reported to be capable carriers of oregano essential oil [19].

Supercritical solvent impregnation (SSI) implies the dissolution of an active compound in a supercritical fluid (scCO_2_) and contact of the supercritical solution with polymer for its functionalization [20,21]. The low viscosity and near-zero surface tension enables the supercritical solution to penetrate the polymer matrix easily [22,23]. Since no organic solvents are used in the impregnation process, high purity final products are obtained by simply depressurizing the system [23]. Furthermore, favorable processing at moderately low temperatures allows polymer modifications without thermal stresses [24] and possible evaporation of volatile active compounds from the impregnated polymer [24,25].

SSI has previously been applied in the production of materials with antimicrobial properties [26,27]. Successful functionalization of synthetic materials such as linear low-density polyethylene (LLDPE) with eugenol [28,29] and thymol [30] in scCO_2_ has been reported. SSI was also applied to the impregnation of PET/PP films with antioxidants extracted from olive leaves [31]. Besides, biodegradable polymers such as poly-lacticacid (PLA) [32] and PLA with 5% of poly(ε-caprolactone) [33] were impregnated with thymol by using the same technique. Furthermore, cellulose acetate films impregnated with thymol by SSI exhibited excellent anti-biofilm activity against *P. aeruginosa* and *S. aureus* [34].

To the best of our knowledge, there are no published reports dealing with the SSI of films based on starch-chitosan blends. The present study aims to investigate the possibility of impregnation of starch and chitosan polymeric biodegradable films with and without zeolite with thymol by scCO_2_. Kinetics of the process, as well as the antibacterial properties of the impregnated films, were studied.

## 2. Results and Discussion

### 2.1. Impregnation in Supercritical Carbon Dioxide

#### 2.1.1. Impregnation of SC Films

In the preliminary experiments, synthesized SC films were exposed to pure CO_2_ at a temperature of 50 °C and a pressure of 30 MPa for 4 h. The results revealed that no extractables were present in the SC films, since their mass remained unchanged after this process. The SSI conditions (35 °C, 15.5 MPa) were adopted from the previous study dealing with the solubility of thymol in scCO_2_ and its impregnation [35].

**Figure 1:**
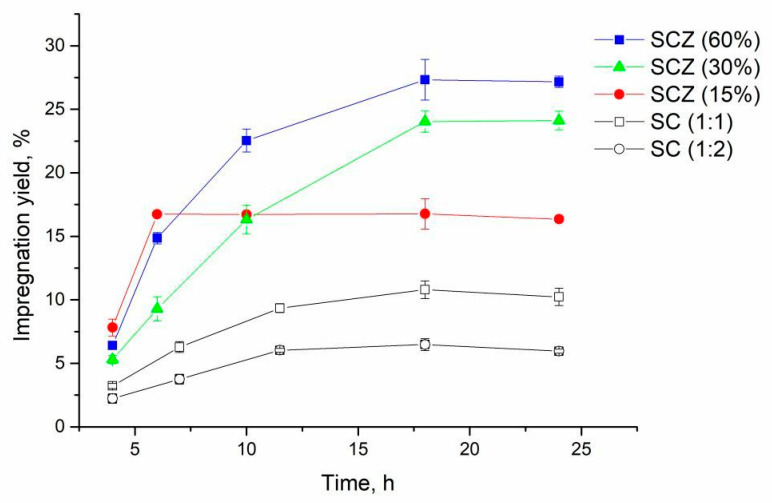
Kinetics for SC and SCZ films with 1:1 and SC films with 1:2 starch/chitosan mass ratios at 35 °C and 30 Mpa. Zeolite content is given in the brackets.

The results showed that no impregnation occurred after 4 h of the experiment at 15.5 Mpa in the case of both films with starch/chitosan mass ratios of 1:1 and 1:2. Therefore, subsequent experiments were performed at a pressure of 30 Mpa. Obtained results are presented in Figure 1.

The SSI of SC films with the starch/chitosan mass ratio of 1:1 was faster compared to the films with the 1:2 mass ratio, whereby the highest impregnation yields of 10.80% and 6.48% respectively were obtained after 18 h of the impregnation process. This can be explained by a lower affinity of thymol for the polymer with a decreased amorphous phase [36]. It was previously reported that the presence of chitosan in an SC blend in amounts higher than 60% resulted in increased crystallinity of the polymer [37].

However, the SCT film with the highest thymol content (10.80%) in our study showed no effect against the tested strains. Therefore, the incorporation of zeolite into the films was highly desirable to improve their loading capacity for thymol and thus indirectly to improve their antibacterial potential. Considering the higher loading capacity of 1:1 SC film, this starch-to-chitosan ratio was chosen for further investigations.

#### 2.1.2. Impregnation of SCZ Films

Kaya et al. reported the high efficiency of the clinoptilolite type natural zeolites in adsorption of oregano essential oils where thymol was the abundant compound [19]. Similarly, our study revealed a high pure zeolite affinity towards thymol, whereby the impregnation yield of 40.7% was obtained after 18 h of the SSI (35 °C, 30 MPa). Based on this result, zeolite was added into the SC blend. The addition of zeolite had a significant effect on the SSI of the SCZ film (Figure 1), increasing its loading capacity for thymol. When 15% of zeolite was added to the film, the obtained impregnation yield increased up to 16.7% after 6 h of the impregnation. This was the maximum film loading since a further increase in the impregnation time did not affect the achieved yield (Figure 1). Increasing the amount of zeolite in the SCZ film to 30% provided thymol loading of 24.1% after 18 h, while the highest impregnation yield for SCZ with 60% of zeolite was 27.3%. As in the case of the impregnated SC films, slight desorption of thymol from the impregnated SCZ polymers with 30 and 60% of zeolite occurred after 24 h of experiment. For the film with a lower amount of zeolite (15%), this process started already after 10 h.

To confirm the gravimetrically determined amount of thymol, SCZT film with 28.4% of thymol was treated with ethanol, and UV-Vis spectroscopy was applied to assess thymol concentration. The deviation in the results obtained by the two methods (gravimetrical and UV-Vis) was minor (0.28%).

### 2.2. Fourier Transform Infrared Analysis

**Figure 2:**
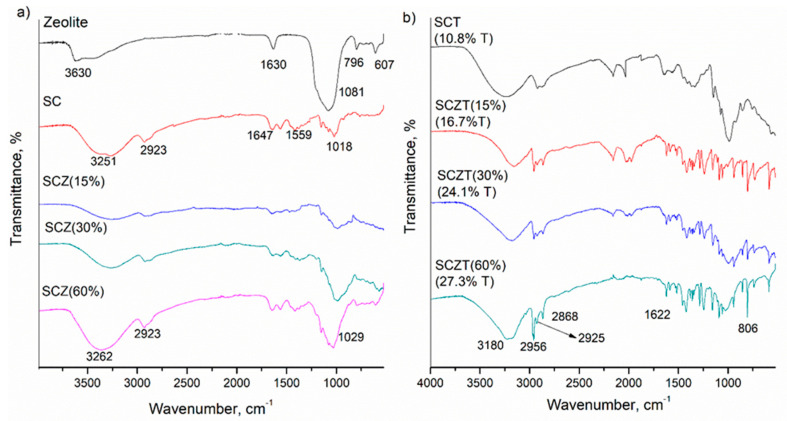
Spectra of the (**a**) control SC and SCZ films and (**b**) thymol impregnated films.

ATR-FTIR was used to analyse the control samples (zeolite, SC, and SCZ) and impregnated films (Figure 2). The amount of impregnated thymol was 10.8% for SC film and 16.7%, 24.1% and 27.3% for SCZ with 15%, 30% and 60% of zeolite, respectively.

FTIR spectrum of the control SC sample reveals characteristic bands, which fit well with the literature data. The broad bands between 3377 cm^−1^ and 3251 cm^−1^ are assigned to OH stretching of the hydroxyl group, which overlaps with N-H stretching vibrations [38]. The band at ~2923 cm^−1^ corresponds to C-H stretching vibrations [38,39]. The band at 1647 cm^−1^ originates from C=O stretching of amide I [38,40]. The appearance of the amino peak at 1559 cm^−1^ indicated that interactions were present between the amino groups of chitosan and the hydroxyl groups of starch [39,41]. Peaks present in the wavenumber region of 1000~1100 cm^−1^ are assigned to C-O stretching vibrations of alcohol groups of both starch and chitosan [42].

The broad band at 3630 and 1630 cm^−1^ in the FTIR spectrum of zeolite can be attributed to the vibrations of water molecules [43,44], while broad band caused by the asymmetric stretching vibration of the Si–O and Al–O bonds from zeolite’s alumosilicate framework is evident at 1081 cm^−1^ [43]. With the addition of zeolite to SC films (Figure 2a), the intensity of the bands in the region of 1000~1100 cm^−1^ increased due to the presence of Si–O and Al–O groups of zeolite, which appears almost at the same frequency as C–O stretching vibrations observed at SC films, suggesting the incorporation of zeolite in the polymer matrix [45].

As expected, FTIR analyses confirmed the presence of thymol on the surface of the impregnated SC and SCZ samples. A broad band in the wavenumber region of 3236–3136 cm^−1^ originated from phenolic OH stretching [46]. The peaks at 2957 cm^−1^ and 2868 cm^−1^ are assigned to stretching vibration of CH_3_ group [35,47]. The band at 1621 cm^−1^ is attributed to C-C stretching of the benzene ring [46]. The bands between 1422 and 1311 cm^−1^ correspond to OH deformation and C-O stretching vibrations [48]. The bands at 1360 and 1345 cm^−1^ are assigned to OH bending and OH deformation vibrations of phenol [46,48]. The band at 806 cm^−1^ corresponds to the out-of-plane aromatic C-H wagging vibration [35,49].

### 2.3. Mechanical Properties of the Films

Mechanical properties of films aimed for application as a food packaging material are essential. Tensile strength is related to the mechanical resistance of films, Young’s modulus relates to the flexibility, and deformation at fracture refers to the capacity of film to extend before breaking. Table 1 presents the values of the tensile strength (σ_T_), Young’s moduli of elasticity and deformation at fracture (E), and elongation to break (ε) of the control samples (SC, SCZ) and impregnated samples.

Amounts of the impregnated thymol were 10.8% for SCT film and 16.7%, 24% and 27% for SCZT films with 15%, 30% and 60% of zeolite, respectively.

The amounts of zeolite in SC samples are given in brackets. SC and SCT samples were found to be the most capable of extending in plastic deformation. Both SC and SCT exhibited the highest tensile strength (10.2 MPa), while E values of SC and SCT samples were 203 MPa and 449 MPa, respectively. Similar values of the tensile strength of starch-chitosan blends were previously reported [50]. Namely, the σ_T_ values were 10.61 and 9.27 MPa, for blends of chitosan-potato starch and chitosan-cassava starch, respectively. The results related to σ_T_ and ε obtained in this study are in accordance with the study of Bonilla et al. [51], where the change in tensile strength of chitosan films with different essential oils was not obvious, while the elongation at break decreased with the oil addition.

The introduction of zeolite into the biocomposite films decreased the value of σ_T_ by 10–38% and increased the deformation at fracture up to 106% compared to SC film. According to the literature, the amount of incorporated zeolite considerably affects the mechanical properties of the polymer [52]. Small to moderate amounts of zeolite enhanced the mechanical strength of the chitosan matrix used for the fabrication of the hybrid membranes. However, increased zeolite amounts (>20%) caused a reduction in mechanical strength due to the formation of too many interfacial voids at the interface of chitosan and zeolite [52]. In the present study, the addition of 15% of zeolite to SC films caused only an insignificant reduction of σ_T_ from 10.2 to 9.1 MPa, while a slightly higher reduction of the tensile strength (7.8 MPa) was noticeable in the case of the SC samples with 30% of zeolite. The increased amount of zeolite (60%) caused a further reduction of σ_T_ to 6.6 MPa, and therefore this film was not considered as a candidate for food packaging in forthcoming tests. Elongation at break of SCZ films was lower compared to the SC films. The increase in stiffness of the films due to the zeolite addition could be related to the interaction between dispersed zeolite and starch and chitosan chain segments, which reduce chain mobility and hence increase rigidity [53]. Impregnation of SCZ polymers with thymol had a negligible effect on the tensile strength and elongation to break, while the deformation at fracture increased in all cases. Similarly, Peng and Li reported that thyme essential oil did not significantly affect the mechanical response of the wheat starch–chitosan films [54].

### 2.4. Scanning Electron Microscopy (SEM) of the Films

**Figure 3:**
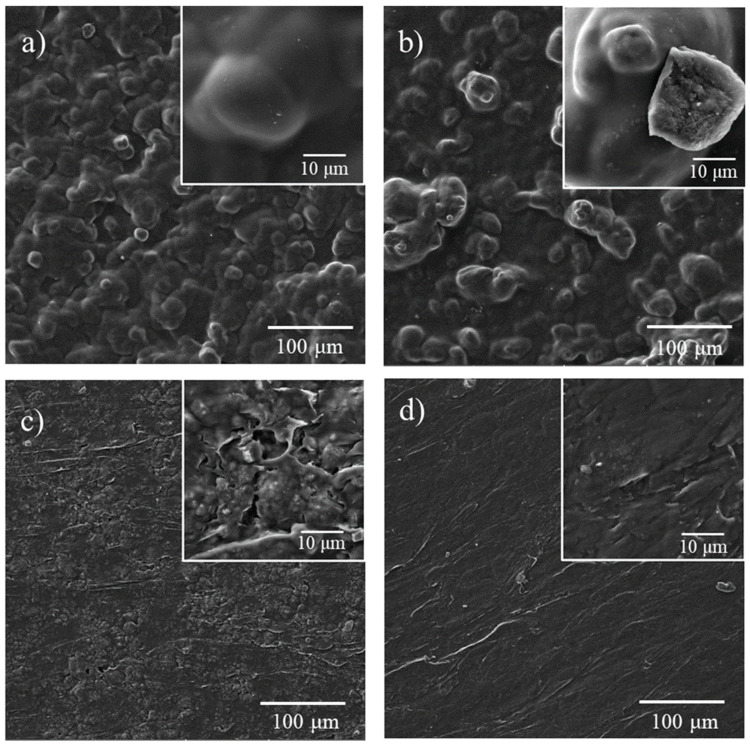
SEM images of the (**a**) control SC film, (**b**) SCT film with 10.08% of thymol, (**c**) control SCZ (30%) and (**d**) SCZT (30%) film with 24% of thymol.

SEM images of SC and SCZ films before and after the SSI with thymol are presented in Figure 3. SC film with a starch to chitosan mass ratio of 1:1 showed a characteristic pebble type structure [55] (Figure 3a). As can be seen in Figure 3b, the SSI did not affect the morphology of the SC film remarkably. This is in agreement with the results of the mechanical analysis performed in this study. Incorporation of zeolite into the SC composite led to a more rigid structure with the presence of voids and cracks (Figure 3c). After the SSI with thymol, the surface of the SCZ sample got a slab-like texture, probably due to the presence of a considerable amount of thymol, which could have induced slight polymer melting (Figure 3d).

### 2.5. Thermogravimetric and Derivative Thermogravimetric Analysis

**Figure 4:**
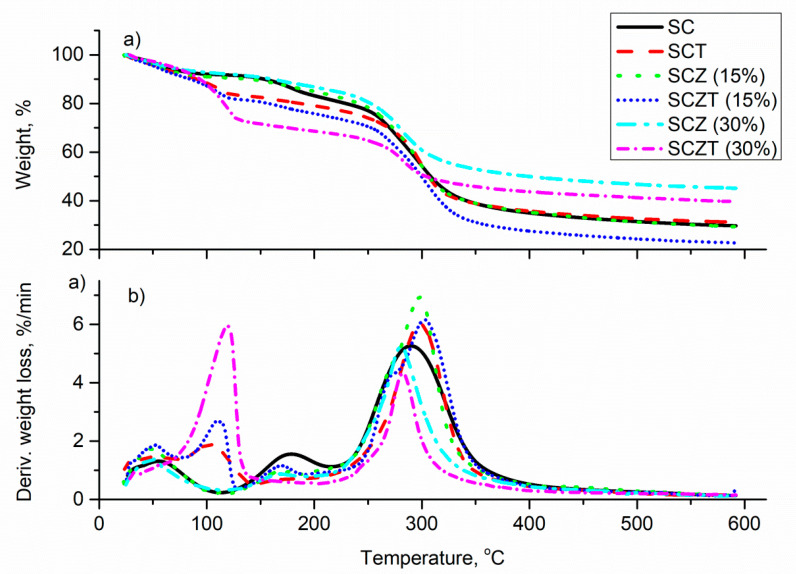
(**a**) TGA and (**b**) DTG curves of the prepared films.

TGA-DTG curves of SC and SCZ films before and after the impregnation with thymol are shown in Figure 4. As can be seen, non-impregnated SC and SCZ films exhibited a similar thermal profile with three decomposition stages. The initial weight loss at 52–60 °C corresponds to the loss of physically adsorbed water molecules, while the second step can be ascribed to the loss of strongly hydrogen-bonded water and glycerol used for the film preparation [56]. The most pronounced weight loss, followed by a peak at DTG with the maximum at ~290–300 °C, corresponds to complex decomposition processes of starch and chitosan, including the depolymerization of chains, dehydration and deamination of the saccharide rings [56,57]. After impregnation of thymol into SC and SCZ films, the peak centred around 103–108 °C can be seen at the DTG curves. Corresponding weight losses ascribed to thymol at TGA curves were around 24.5%, 14.5% and 10% for SCT, SCZT (15%) and SCZT (30%) samples, respectively. These values were in accordance with previously determined impregnation yields. The slight shift of the films’ degradation temperature to somewhat higher values indicates an improved thermal stability of impregnated films, which is explained by interactions between the polymer chains and the molecules of thymol [33,56]. Results showed that films impregnated with thymol are stable up to 100 °C, and are thus suitable for safe application in the food packaging industry.

### 2.6. Water Vapor Permeability of the Films

The results of the barrier properties’ analysis are presented in Table 2. As can be seen, the higher the zeolite content, the higher the WVP. Such an effect could be explained by an increased distance between polymer chains inside the network due to the addition of zeolite. This made the polymer matrix less compact and favourable to the adsorption/desorption of water molecules. The WVP of the SC film obtained in this study (1.78 × 10^−11^ gm^−1^s^−1^Pa^−1^) was in the range of the published result by Acter et al. (2.84 × 10^−11^ gm^−1^s^−1^Pa^−1^) [58]. It was previously reported that the inclusion of hydrophobic components to biopolymer films resulted in a decreased WVP due to the increased hydrophobicity of the composites [6]. This was in agreement with the results obtained in this study since the addition of thymol slightly decreased the permeability of impregnated films compared to the controls. The lowest WVP was observed in the case of the SCT film (1.70 × 10^−11^ gm^−1^s^−1^Pa^−1^). The addition of zeolites increased the WVP value.

Amounts of the impregnated thymol were 10.8% for SC film, and 16.7% and 24% for SC films with 15% and 30% of zeolite, respectively.

Li et al. [59] reported the barrier properties of gelatine films blended with lecithin and thymol with obtained WVP values between 7.19 ± 0.18 × 10^−11^ and 10.09 ± 0.13 × 10^−11^gm^−1^s^−1^Pa^−1^. Medina et al. [60] investigated the barrier properties of chitosan-quinoa films containing chitosan-thymol nanoparticles with obtained WVP values of 0.34 ± 0.05 × 10^−8^gm^−1^s^−1^Pa^−1^. Another study [61] of basil seed gum films impregnated with oregano essential oil reported WVP values in the range of 3.7–4.3 × 10^−8^gm^−1^s^−1^Pa^−1^.

### 2.7. Release Kinetics

The release of the active substance from the carrier occurs in three steps: diffusion of surrounding liquid into the polymer, polymer matrix relaxation and active component dissolution and diffusion into the outer solution [62]. In this study, distilled water was used as a model medium for the release of thymol from prepared films as one of the aqueous food simulants, according to European Standard EN 13130-2005 [7], to evaluate the effect of the moisture from food systems.

**Figure 5:**
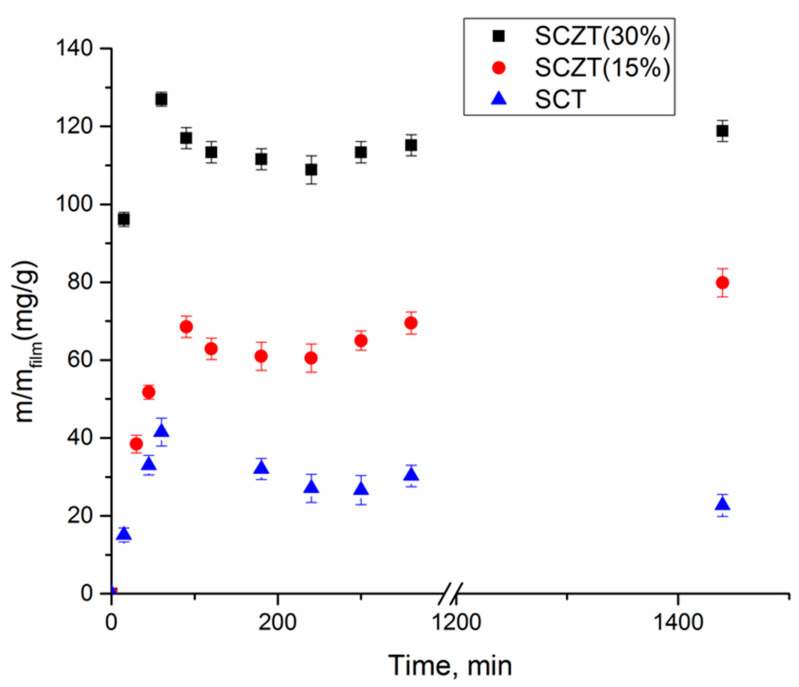
Thymol release kinetics from SCT film with 10.08% of thymol, SCZT (15%) with 16.7% of thymol and SCZT (30%) with 24% of thymol into distilled water.

Similar release profiles were obtained with all tested films, with the fast release rate detected in the first hour of the contact with water (Figure 5).

The effect can be explained by the easy and rapid diffusion of water molecules into the polymer matrix, causing the film hydration and network weakening, thus producing an open structure and increasing thymol release [62]. High water sensibility of both starch and chitosan contributed to the observed rapid release. Moreover, glycerol, which was used as a plasticizer during the film preparation, caused the disruption of intermolecular forces between polymer chains, thus opening the matrix structure and enabling the thymol uptake [63]. After one hour, the equilibrium was reached for all tested films, as the amount of released thymol remained constant in the next 24 h. For the SCZT (30%) with 24.1% of thymol, the amount of thymol released at equilibrium was 118.82 mg/g_film_, representing 35.7% of the initially loaded substance. The amount of thymol released after 24 h from SCZT (15%) with 16.7% of thymol was 79.85mg/g_film_ (45.5% of the initial load). In the case of the SCT film with 10.8% of thymol, after 24 h, the amount of 22.68 mg/g_film_ was released, corresponding to 25.0% of the initially loaded quantity. The released amounts from all films are well below the saturation value of thymol in water (900 mg/L) [33]. Thus, thymol’s solubility was not the limiting factor to the mass transfer into the surrounding medium. This implies that interactions between the carrier and phenolic groups of thymol caused its retention in the matrix in high amounts.

Release of thymol from different carriers has been previously reported. The release profile and the amount of thymol released depend on the carrier’s morphology, thymol loading, nature of the medium, and its solubility in the medium and carrier material. The released amount of thymol in distilled water from poly(d,l-lactic-co-glycolic acid) (PLGA) with 6.62% loading was around 4.8 mg/g after 24 h [64]. In another study [21], the sample of cellulose acetate with the highest thymol loading (63.84%) reached the highest value of released thymol of around 400 mg/g. Release of thyme extract polyphenols from starch/chitosan blend films in water after 24 h was 7.7 mg/g polymer, or 17% of the total polyphenols incorporated [65]. The high retention of polyphenolic compounds in the film was attributed to their strong interactions with chitosan chains.

**Figure 6:**
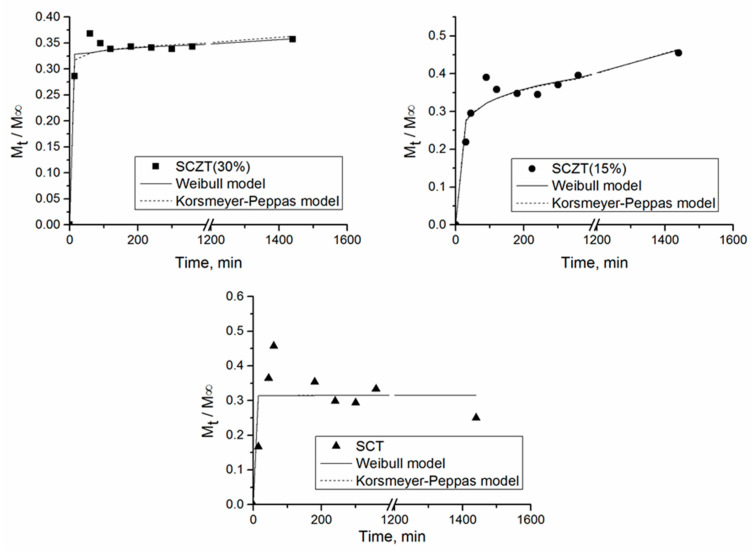
Relative mass of thymol released from SCT film with 10.08% of thymol, SCZT (15%) with 16.7% of thymol and SCZT (30%) with 24% of thymol.

To study the release mechanism, the experimental data were correlated with two kinetic models (Equations (3) and (4)). Korsmeyer–Peppas power law model [66] and Weibull function [67] were applied to simulate the release kinetics of thymol from SCT and SCZT films (Figure 6). The determined values of the model parameters are presented in Table 3.

As can be seen from Figure 6 and Table 3, the models fitted the data with similar accuracy. Both models could be used to describe the kinetics of thymol release from SCZT films (R^2^ = 0.936–0.975), while a lower agreement of the models with the experimental data was obtained for the release of thymol from the SCT film (R^2^ = 0.63). The value of the diffusional exponent, *n*, of the Korsmeyer–Peppas model was lower than 0.5, implying that a quasi Fickian diffusional mechanism controlled thymol release from the prepared films [65].

### 2.8. Antibacterial Activity

**Figure 7:**
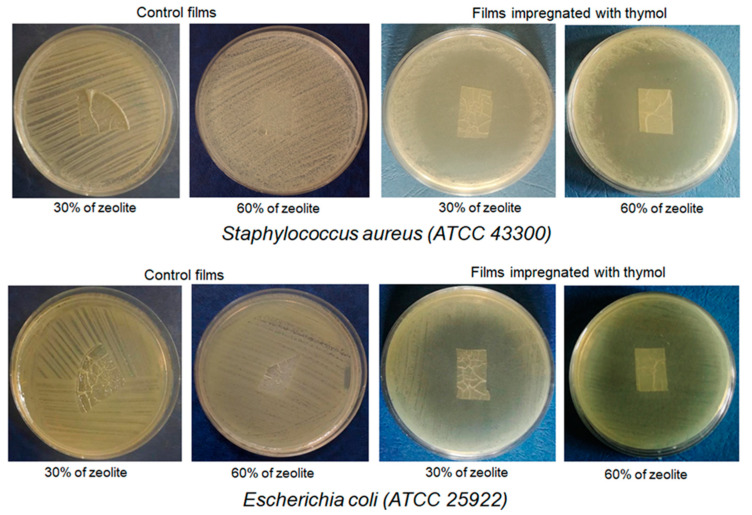
Antibacterial activity of the control and SCZT films with 24% of thymol.

The results of the antibacterial analyses are presented in Figure 7.

No inhibition zones were recorded around the neat SC and SCZ (Figure 7), with bacterial growth detected even beneath the films. Similarly, no inhibition zones were detected around SCT films impregnated with 10.80% of thymol. SCZT films with 15%, 30% and 60% of zeolite and with approximately 17%, 24% and 27% of thymol, respectively, showed significant antibacterial activity with inhibition zones of >50 mm for both *E.coli* ATCC 25922 and MRSA ATCC 43300. SCZT film with 60% of zeolite and 27.3% of thymol showed stronger activity, i.e., the greatest inhibition zone of ˃70 mm in *E.coli* ATCC 25922. The applied diffusion method (as a modification of the antibiotic disk diffusion test) can be categorized exclusively as a screening qualitative test that will provide information on whether thymol is released from the film on the surface of the culture medium, whether it diffuses and whether it retains its antimicrobial properties [68]. More precise data on the potency of antimicrobial agents of natural origin, including thymol, are obtained by determining the MIC (minimum inhibitory concentration) values in the so-called dilution methods (broth micro and macrodilution, agar dilution) [68]. In the case of impregnated films (which are a mixture of different polymers), the determination of the MIC is impossible. The main obstacles are solid consistency of water-insoluble films, as well as the presence of insoluble zeolites and thymol (the method is performed exclusively in water-based media). Individual thymol MIC can be determined by dissolving thymol in DMSO, but the use of DMSO in impregnated materials, due to the complexity of their composition, is not possible. Through the obtained results, it can be concluded that thymol is an excellent choice for impregnation of functional-active packaging films for the food industry. Firstly, unlike most other natural bioactive molecules/extracts that usually show action only against Gram-positive bacteria, thymol has good activity against both Gram-positive and Gram-negative bacteria at the same time as having good activity against viruses and parasites [1,3,10,34,46]. In addition, thymol is excellently released from the tested films and diffuses excellently over the surface of the nutrient medium while retaining its strong antibacterial activity. And finally, the flavouring properties of thymol are similar to the commonly used culinary plants in which it is most abundant (for example, thyme).

Obtained antibacterial activity of SCZT (30%) film with 24% of loaded thymol, coupled with better mechanical properties compared to the film with 60% of zeolite, qualifies this film as a promising candidate for further investigation and optimization.

## 3. Materials and Methods

### 3.1. Materials

High molecular weight chitosan (310000–375000 g/mol, 75–85% deacetylated) was purchased from Sigma-Aldrich Chemie GmbH, (Steinheim, Germany), and corn starch (amylose content 28%) from Jabuka (Pancevo, Serbia). Commercial CO_2_ (purity 99%) was supplied by Messer–Tehnogas (Belgrade, Serbia), sodium chloride (p.a. grade) from Sigma-Aldrich Chemie GmbH (Steinheim, Germany), and ethanol (99.9%) from Merck (Millipore, Darmstadt, Germany). The natural zeolite tuff, which contained about 70 wt.% of clinoptilolite (quartz and anorthite were major impurities), was obtained from Nizny Hrabovec, Slovakia.

### 3.2. Preparation of Zeolite Sample

The particle size of the natural zeolite tuff was in the range of 0.063–0.1 mm, and the cation exchange capacity was 118 mmol M^+^/100g. The sample used for the SCZ film preparation (1.0 g) was pre-treated with 100 cm^3^ of 2.0 mol dm^−3^ solution of NaCl to improve the tuff’s exchange capacity [69,70]. The suspension was magnetically stirred for 24 h at 25 °C and then separated. The purified natural zeolite was washed with distilled water and dried in an oven overnight at 105 °C. Before the application in the films’ synthesis, zeolite particles were milled to a size of approximately 1µm using a planetary ball mill Retsch PM 100.

### 3.3. SC and SCZ Films Preparation

Chitosan was dissolved in a 2% (*w*/*v*) acetic acid solution to yield a 1% (*w*/*v*) chitosan suspension. The suspension was stirred using a magnetic stirrer until the clear solution was obtained. The 1% (*w*/*v*) starch solution was prepared by dispersing the starch in distilled water and heating the mixture on a hotplate for 10 min at 90 °C with stirring. Starch-chitosan films were prepared by mixing the starch solution (1% *w*/*v*) with the chitosan solution (1% *w*/*v*) in mass ratios of 1:1 and 1:2, respectively. Glycerol (25% *w*/*w* of the total solid weight) was added to the mixture. The mixture was stirred using a magnetic stirrer until it had cooled to the room temperature of 22 °C. Afterwards, the obtained solution was cast into the plastic molds (5 cm diameter). After drying at room temperature for 24 h, the films were additionally dried in an oven at 50 °C for 24 h.

In order to obtain SCZ films, a different amount of zeolite (15, 30 and 60% (*w*/*w*) based on the total solid weight) was added during the mixing step of chitosan and starch solutions. Prior to casting the solution into plastic molds (5 cm diameter), the suspension was vigorously stirred (15,000 rpm) for 15 min using an Ika Ultra-Turrax disperser (Staufen, Germany). The subsequent steps of drying were maintained as previously described.

### 3.4. Supercritical Impregnation

SSI of the SC and SCZ films with thymol was performed in a high-pressure view chamber (Eurotechnica GmbH, Bargteheide, Germany), using a static method, as previously described [20,35]. Thymol was placed in a glass container at the bottom of the vessel. The film was placed in the porous basket above the container with thymol. PTFE-coated fiberglass fabrics were placed below the film to prevent possible splashing of thymol onto the surface of the film during the decompression. In order to assure the excess of thymol, the film (SC, SCZ) to thymol mass ratio was set to 0.025 ± 0.005. After the process temperature of 35 °C had been reached, CO_2_ was introduced into the system by a high-pressure pump (Milton Roy, Pont-Saint-Pierre, France) until the desired pressure was reached (15 or 30 MPa). The system was kept at a constant pressure and the temperature for the desired time was in the range of 4–24 h with subsequent decompression at the rate of 1.5 MPa/min. The mass of the impregnated thymol (*m_t_*) was determined gravimetrically as the mass difference of the sample after and before the impregnation process (quantified on an analytical scale with an accuracy of ±0.0001 g). The impregnation yield (*I*) was calculated as the mass ratio of the impregnated thymol and impregnated film (*m_if_*) multiplied by 100% (Equation (1)).

(1) $$I\;\left(\%\right)=\frac{m_{t}}{m_{if}}\times 100\%$$

Impregnated films were denoted as SCT and SCZT (X%), with X denoting the amount of zeolite added.

### 3.5. Re-Extraction of the Impregnated Samples

Thymol was re-extracted from the impregnated films with ethanol to check the impregnation yield obtained gravimetrically. The sample was cut into small pieces (~3 × 3 mm) and suspended in an appropriate volume of ethanol (100 mL) to obtain the final thymol concentration of approximately 0.2 mg/mL. The suspension was magnetically stirred over the night at room temperature to provide complete desorption of thymol from the impregnated polymer.

Thymol concentration after the re-extraction was determined at 274 nm [21] using a UV-Vis spectrophotometer (Shimadzu 1800, Kyoto, Japan).

### 3.6. Characterization of the Samples

#### 3.6.1. FTIR Analysis

Fourier-transform infrared (FTIR) spectra of thymol-impregnated and control films were recorded in the ATR mode using a Nicolet™ iS™ 10 Spectrometer (Thermo Fisher SCIENTIFIC, Darmstadt, Germany) with a resolution of 4 cm^−1^ at wavenumbers in the range of 500–4000 cm^−1^.

#### 3.6.2. SEM Analysis

The surface morphology of the SC and SCZ samples before and after the SSI with thymol was analyzed by field emission scanning electron microscopy (FESEM, Tescan Mira3 FEG, Brno, Czech Republic). The samples were coated with a thin layer of gold before the analysis.

#### 3.6.3. TGA-DTG Analysis

Thermal properties of the prepared films were investigated by thermogravimetric and derivative thermogravimetric analysis (TGA-DTG) using a SDT Q600 simultaneous TGA-DTA instrument (TA Instruments, New Castle, DE, USA). The samples were heated in a standard alumina sample pan from room temperature to 600 °C at a heating rate of 10 °C min^−1^ under a nitrogen atmosphere with a flow rate of 100 cm^3^ min^−1^.

#### 3.6.4. Mechanical Analysis

The mechanical properties of films before and after the impregnation were tested with an Instron 1185 universal testing device at room temperature. Tensile testing of specimens was performed at 2 mm/min crosshead speed. Module (E) was calculated as a slope of the initial linear portion of the stress–strain curve. Tensile strength (σT) as tensile stress at break and percent elongation at break (ε%) were calculated from tension data. Five or more specimens of each formulation were tested, and average values were reported.

#### 3.6.5. Water Vapor Permeability

Water vapor permeability (WVP) tests were performed gravimetrically according to the standardized methodology [37,71]. Measurements were carried out at 75% relative humidity (RH) at 25 °C in the desiccator (Star desiccator, Bola, Grünsfeld, Germany). The samples with an exposed area of 12.56 cm^2^ were sealed over a circular opening of aluminium permeation cells filled with anhydrous sodium chloride to provide 0% RH inside the cell and placed inside the desiccator. Saturated sodium chloride was used to provide an atmosphere of 75% RH in the desiccator. Due to the moisture gradient, water vapor penetrates through the tested samples towards the inside of the cells. Water vapor uptake by anhydrous sodium chloride results in a weight increase of the cells. The cells’ weight change was determined twice a day for five days, using an analytical scale with an accuracy of ±0.0001 g. Tests were performed in triplicates. The standard deviation was ±10%.

Changes in the weight of the cells were plotted with respect to time, and the linear least-square method was used for the calculation of the parameters given by Equation (2) [72]:

(2) $$WVP=\frac{L\cdot WVTR}{A\cdot ∆p}$$

where *WVTR* is the water vapor transmission rate of films (gs^−1^), *L* is the average thickness of the film (m), A is the permeation area (m^2^) and Δp is the difference in water vapor pressure between the two exposed sides of the film (Pa).

### 3.7. Release Kinetics

A kinetic study on thymol release from the impregnated SCT, SCZT (15%) and SCZT (30%) films in deionized water was performed using a UV-Vis spectrophotometer (Shimadzu 1800, Kyoto, Japan) at 274 nm. Film samples (0.0045 ± 0.0005 g) were immersed in 100 mL of distilled water at 25 °C without stirring. At pre-determined periods, an aliquot (3.5 mL) of the solution was taken, analysed and returned into the release medium. Release experiments were carried out in duplicate for 24 h, i.e., until no change in the released amount of thymol was detected. Thymol concentration was calculated using a previously determined calibration curve. Experimental results were given as released mass of thymol per g of film plotted against time. Two kinetic models, Korsmeyer-Peppas (Equation (3)) and Weibull (Equation (4)), were used to fit the experimental data:

(3) $$\frac{M_{t}}{M_{\infty }}=k\cdot t^{n}$$

(4) $$\frac{M_{t}}{M_{\infty }}=1-\exp(-a\cdot t^{b})$$

where *M_t_* is the amount of thymol released at time *t*, *M_∞_* is the starting amount of thymol incorporated in the film; *k* is kinetic constant; *n* is the diffusional exponent; and *a* and *b* are constants. The nonlinear regression module of Polymath Educational 6.10 software package (Willimantic, CT, USA) was used to determine parameters of the models used.

### 3.8. Antibacterial Activity

The study of antibacterial activity of SCT and SCZT films (starch/chitosan molar ratio 1:1, impregnated with thymol) was conducted on Methicillin-resistant *Staphylococcus aureus* MRSA ATCC 43300 and *Escherichia coli* ATCC 25922. An agar diffusion test was used as a qualitative method to determine the antimicrobial activity of the samples with the thymol content of 10.80% for SCT and ~17% and ~24% for SCZT, with a different amount of zeolite added. Agar diffusion testing was carried out as previously described [68,73]. The direct colony suspension method in sterile saline was used to prepare an inoculum of each strain in order to achieve a density of 0.5 McFarland turbidity standard (approximately 1–2 × 10^8^ CFU/mL). Agar diffusion was performed on Mueller Hinton agar (Becton Dickinson, Heidelberg, Germany). Each SC and SCZT film impregnated with thymol was cut to approximately equal pieces, and one of the pieces was placed on the surface of the inoculated agar plate. The plates were incubated at 37 °C for 24 h. The non-impregnated SCT and SCZT films were used as controls. The results were recorded as the presence or absence of the inhibition zone around the films. The zones of inhibition appeared as clear zones without bacterial growth around SCT and SCZT films.

## 4. Conclusions

Starch-chitosan films with and without zeolite were synthesized and impregnated with thymol in scCO_2_. The SSI performed at 35 °C, and 30 MPa efficiently incorporated thymol into both SC and SCZ films. A higher impregnation yield (10.80%) was obtained in SC films with a starch/chitosan mass ratio of 1:1 compared to the ratio of 1:2 (6.48%). The introduction of zeolite into the SC blend (1:1 mass ratio) notably improved the loading capacity of films, with up to 27.3% of loaded thymol in the film with 60% zeolite. The FTIR analysis confirmed the presence of thymol on the surface of the films. Young’s moduli of elasticity increased with the zeolite addition, while tensile strength decreased. The addition of zeolite increased the film’s water vapor permeability, while the posterior impregnation had an opposite effect. SCZ films impregnated with thymol (24% and 27%) showed a significant antibacterial effect against MRSA ATCC 43300 and *E. coli* ATCC 25922, which proved that thymol is being released from the film in the surrounding culture medium and that its antibacterial activity has been preserved after the impregnation in polymers. It can be concluded that the produced starch-chitosan film with 30% of zeolite impregnated with thymol has the potential to be considered for food packaging systems. However, further investigations on the possible cytotoxic effects of the synthesized films on human cell lines are needed.

## Figures and Tables

**Table 1:** Tensile strength (σ_T_), Young’s moduli of elasticity and deformation at fracture (E) and elongation to break (ε) of the control and thymol impregnated SC and SCZ films.

Sample	σ_T_ (MPa)	E (MPa)	ε (%)
SC	10.2 ± 1.8	203 ± 19	20.1 ± 6.9
SCT	10.2 ± 2.3	449 ± 52	10.0 ± 2.3
SCZ (15%)	9.1 ± 2.1	419 ± 93	2.5 ± 0.3
SCZT (15%)	9.7 ± 0.9	466 ± 65	3.1 ± 0.2
SCZ (30%)	6.3 ± 0.7	321 ± 55	3.7 ± 0.7
SCZT (30%)	7.8 ± 0.6	447 ± 63	3.1 ± 0.4
SCZ (60%)	6.6 ± 1.1	380 ± 68	2.8 ± 0.8
SCZT (60%)	6.1 ± 1.3	623 ± 130	1.4 ± 0.2

**Table 2:** Water vapor permeability of the control and impregnated films.

Sample	WVP × 10^11^ (gm^−1^s^−1^Pa^−1^)
SC	1.78 ± 0.12
SCT	1.70 ± 0.14
SCZ (15%)	2.21 ± 0.21
SCZT (15%)	1.97 ± 0.18
SCZ (30%)	2.71 ± 0.20
SCZT (30%)	2.54 ± 0.24

**Table 3:** Parameters and correlation coefficients (R^2^) of the release kinetics models.

Film	Korsmeyer–Peppas Equation (3)			Weibull Equation (4)		
	*k*, h^−n^	n	R^2^	a	b	R^2^
SCT	0.314	0.0012	0.630	0.377	0.0015	0.630
SCZT (15%)	0.305	0.132	0.936	0.362	0.170	0.938
SCZT (30%)	0.330	0.029	0.975	0.402	0.030	0.968
